# Supplementary material for: Potential Distribution and the Risks of Bactericera cockerelli and Its Associated Plant Pathogen Candidatus Liberibacter Solanacearum for Global Potato Production
Source: Insects. 2020 May 12;11(5):298. doi: 10.3390/insects11050298 (PMC7291056; doi:10.3390/insects11050298)
Supplement: Supplementary file 1 [file insects-11-00298-s001.pdf]

Supplemental Materials

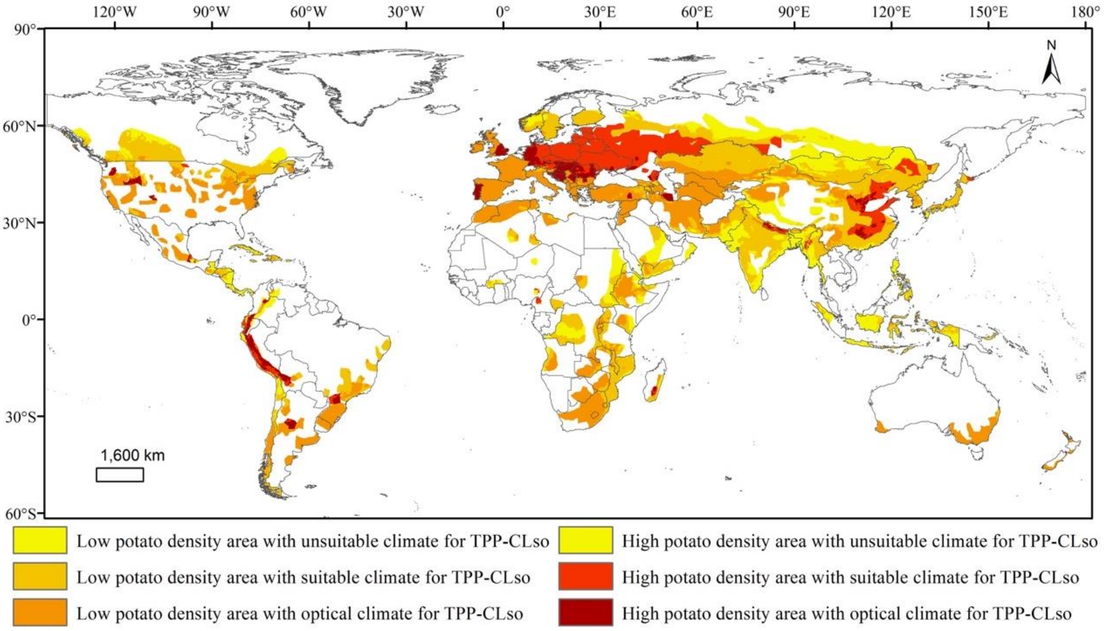

Figure S1. Overlap between global potato cultivation areas and predicted climatically suitable areas for TPP and CLso.
